# The Ferroptosis-NLRP1 Inflammasome: The Vicious Cycle of an Adverse Pregnancy

**DOI:** 10.3389/fcell.2021.707959

**Published:** 2021-08-20

**Authors:** Li Meihe, Gao Shan, Kang Minchao, Wu Xiaoling, An Peng, Wu Xili, Zheng Jin, Dang Huimin

**Affiliations:** ^1^Department of Traditional Chinese Medicine, Second Affiliated Hospital of Xi’an Jiaotong University, Xi’an, China; ^2^Beijing Traditional Chinese Medicine Hospital Affiliated to Capital Medical University, Beijing, China; ^3^Department of Thoracic Surgery, First Affiliated Hospital of Xi’an Jiaotong University, Xi’an, China; ^4^Health Science Center of Xi’an Jiaotong University, Xi’an, China; ^5^Department of Obstetrics and Gynecology, Second Affiliated Hospital of Xi’an Jiaotong University, Xi’an, China; ^6^Department of Renal Transplantation, First Affiliated Hospital of Xi’an Jiaotong University, Xi’an, China

**Keywords:** ferroptosis, HTR-8/SVneo, oxidative stress, NLRP1 inflammasome, pathological pregnancy

## Abstract

One of the hallmarks of placental dysfunction is the increase of oxidative stress. This process, along with the overexpression of the inflammasome, creates a downward spiral that can lead to a series of severe pregnancy complications. Ferroptosis is a form of iron-mediated cell death involving the accumulation of reactive oxygen species, lipid peroxides. In this study, the rats’ model of oxidative stress abortion was established, and hydrogen peroxide (H_2_O_2_) was used to establish a cellular model of placental oxidative stress. RNAi, western blot, and immunofluorescence were used to evaluate the expression of specific markers of ferroptosis and the expression of the inflammasome in placental trophoblast cells. We observed excessive levels of ferroptosis and inflammasome activation in both rats’ model and placental trophoblast cell model of oxidative stress. When the NLRP1 inflammasome was silenced, the expression levels of GSH and Glutathione peroxidase 4 (GPX4) were increased, while the expression levels of transferrin receptor 1 (TFR1), acyl-CoA synthetase long-chain family member 4 (ACSL4), Superoxide dismutase (SOD), and Malondialdehyde (MDA) were decreased. However, when an NLRP1 activator was applied, we observed the opposite phenomenon. We further explored the mechanisms underlying the actions of ferroptosis to inflammasomes. The expression levels of NLRP1, NLRP3, IL-1β, and caspase-1 were positively correlated with the ferroptosis following the application of ferroptosis inhibitor (ferrostatin-1) and ferroptosis activator (erastin). The existence of ferroptosis was demonstrated in the oxidative stress model of placental trophoblast cells; the results also indicate ferroptosis is linked with the expression of NLRP1 inflammasome. These findings may provide a valuable therapeutic target for the pathogenesis of pregnancy-related diseases.

## Introduction

The placenta is the main organ by which a mother can delivers nutrients to her fetus. It can also help to protect the fetus from certain foreign biomolecules, infections, and maternal diseases. Therefore, the normality of this organ is essential for a normal physiological pregnancy and for the delivery of a healthy baby (Gude et al., 2004). In addition, the normal functionality of trophoblast proliferation and invasion is essential for blastocyst implantation, placental formation, and an appropriate mother–fetus relationship (Gude et al., 2004).

Oxidative stress reflects an imbalance between intracellular reactive oxygen species (ROS) levels and the antioxidant defense systems. There is evidence that placental and systemic oxidative stress may play a role in the pathogenesis of adverse pregnancies, such as threatened abortion and preeclampsia (Ferguson et al., 2017; Aouache et al., 2018; Taravati and Tohidi, 2018; Vaka et al., 2018). The levels of ROS and lipid peroxidase in the placental tissues of patients with preeclampsia are significantly increased; in contrast, levels of antioxidant enzymes, including superoxide dismutase (SOD) and glutathione peroxidase (GSH), are decreased (Wang and Walsh, 1996, 2001; Serdar et al., 2003). Oxidative damage to the placenta leads to inflammation and apoptosis, and the resulting cellular debris is released into the maternal circulation. These placenta-derived factors can act on the maternal endothelium, leading to systemic endothelial dysfunction (Goulopoulou and Davidge, 2015), and ultimately, to a pathological pregnancy. Therefore, reducing placental oxidative stress is a feasible strategy to ensure maternal and fetal health.

Inflammasomes are multiprotein complexes in the cytoplasm that are responsible for the formation of pro-inflammatory molecules (Kaushal et al., 2015; Zhang et al., 2017). Nod-like receptor (NLR) family protein 1 (NLRP1) was the first member of the NLR family to be reported and is an important component of the inflammasome (Masters et al., 2012). The NLRP1 inflammasome consists of NLRP1, which recognizes danger signals or ligands, caspase-1, which is critical for inflammasome activation, and ligand ASC (an apoptosis-related speckle-like protein containing the caspase recruitment domain) (Wang et al., 2015). Inflammasomes activate the protease caspase-1, which decomposes IL-1 into biologically active IL-1β (Hassan and Amer, 2011). Activation of the inflammasome requires the production of ROS because most known inflammasome stimuli trigger ROS production, and treatment with various ROS scavengers is known to prevent inflammasome responses to agonists (Schweneker et al., 2013; Lebel et al., 2014). In our previous studies, we found that in H_2_O_2_-induced oxidative stress, there is a large amount of ROS production, as well as the overexpression of NLRP1 inflammasomes (Li et al., 2020). This suggests that the NLRP1 inflammasome plays an important role in the occurrence and development of adverse pregnancy.

Ferroptosis is a novel, adaptive, and programmed process that leads to regulatory cell death, and was first described in 2012 (Dixon et al., 2012). Ferroptosis is mainly caused by iron-dependent lipid peroxidation damage. This iron, primarily known as Fe^2+^ [ferrous, Fe (II)], is highly reactive and toxic because it contributes to the generation of hydroxyl radicals, which are ROS with strong oxidizing properties (Li et al., 2019). Ferroptosis differs from apoptosis, necrosis, and autophagy with regards to programmed cell death and has been associated with oxidative stress (Hirschhorn and Stockwell, 2019). The need for iron increases significantly during pregnancy to support the growth and development of the fetus. Epidemiological studies have shown that excess iron intake and/or high iron status is detrimental to pregnancy and is associated with pregnancy diseases such as preeclampsia (Zhang et al., 2020). We hypothesized that many of the clinical manifestations of reproductive disorders and pregnancy complications may be due to underlying ferroptosis, characterized by disorders of iron homeostasis, thus leading to excessive levels of ferroptosis.

Previous work established that the NLRP1 inflammasome is involved in the occurrence and development of pregnancy diseases, but whether ferroptosis plays a role in this pathway remains unclear. Furthermore, we do not yet understand the relationships and interactions between the NLRP1 inflammasome and ferroptosis with regards to homeostatic processes in trophoblast cells. Therefore, in the present study, we chose to use H_2_O_2_ to establish an *in vitro* model of oxidative stress in HTR-8/SVneo placental trophoblast cells, using RNAi technology, ferroptosis inhibitors, and ferroptosis activators. Our aim was to verify the existence of ferroptosis and identify the relationship between ferroptosis and the NLRP1 inflammasome in a model of oxidative stress from three aspects. Understanding the oxidative stress caused by poor placental pathological pregnancies could provide a new theoretical basis for pathogenesis and highlight new therapeutic targets.

## Materials and Methods

### Establishment of the Rat Oxidative Stress Abortion Model

12 female Lewis rats (8–10 weeks) and 6 male Lewis rats were obtained from the Experimental Animal Center of Xi’an Jiaotong University (Xi’an, China) and housed in a standardized environment: Temperature 22°C, 50–60% humidity and 12 h light-dark cycle with access to laboratory rodent chow and tap water *ad libitum* under pathogen-free conditions. The rats were randomly divided into two groups. Model group: 8 rats were used to establish the abortion model, were given hydroxyurea solution (hydroxyurea powder dissolved in normal saline at a concentration of 450 mg/kg/d) intragastric administration for 10 days, followed by subcutaneous injection of 0.3 mg/kg/d of epinephrine on the fourth day for 7 days. Control group: 8 healthy non-pregnant rats were enrolled, were given normal saline intragastricly and subcutaneously, with the same time and method as the model group. On the 11th day, male and female rats were mated at 1:2, if vaginal suppositories or sperm were found, it was considered the first day of pregnancy. At 9 am on the third day of pregnancy, head and neck subcutaneous injection mifepristone solution 5 mg/kg once (mifepristone grind powder after dissolved in anhydrous ethanol, then suspended in edible oil solution), a control group of head and neck skin amount of ethanol injection oil solvent. At 9:00 a.m. on the fifth day of pregnancy, each group rats after anesthesia, extract the abdominal aortic blood, stripping of the uterine body (Li et al., 2021).

### Cell Culture

Dr. Charles Graham, of Queen’s University of Canada, kindly provided the HTR-8/SVneo cell line. The cells were cultured in DMEM/F12 (HyClone, United States) with 10% FBS (Gibco, United States), 100 U/mL of penicillin, and 100 mg/mL of streptomycin (Gibco); cells were maintained in a humidified incubator with 5% CO_2_ at 37°C.

### Establishment of an Oxidative Stress Model in HTR-8/SVneo Cells

H_2_O_2_ is a stable peroxide free radical and a known inducer of oxidative stress that can promote apoptosis in primary cultured trophoblast cells (Tang et al., 2014). The HTR-8/SVneo cell line is considered the closest study model to trophoblast cells derived from early human pregnancy (8∼10 weeks) (Graham et al., 1993). The HTR-8/SVneo human trophoblast cell line applied in the present study is more similar to primary trophoblasts and normal human physiological conditions. In our previous work, we successfully established a model of oxidative stress in placental trophoblast cells (Li et al., 2020). Cells were cultured in a 37°C, 5% CO_2_ incubator plated on a six-well culture plate at a density of 1 × 10^5^ cells per well. After 24 h, H_2_O_2_ (Sigma-Aldrich, United States) was added at a concentration of 300 μmol/L to the culture, with a control group (untreated placental trophoblast cells) being set up at the same time. All groups were cultured for an additional 3 h under same conditions.

### Detection of Ferroptosis Expression in HTR-8/Svneo Cells After NLRP1 Silencing and the Overexpression of NLRP1

#### Silencing NLRP1 With RNAi

HTR-8/SVneo cells were divided into three groups: Control, siRNA-NC, and siRNA-NLRP1 groups. The siRNA was processed as described below (Table 1). Two hours before transfection, the cell culture medium was replaced with DMEM/F12 medium without serum. siRNA (10 μL) was diluted with 100 μL of serum-free Opti-MEM (at a concentration of 20 μmol). The mixture was gently mixed with a pipette-gun head and kept at room temperature for 5 min. Before use, Lipofectamine^TM^ 2000 was gently mixed, and then 5 μL of Lipofectamine^TM^ 2000 was diluted in 100 μL of Opti-MEM and left to stand for 5 min at room temperature. Lipofectamine^TM^ 2000 and plasmid diluent (total volume of 200 μL) were then mixed gently and left to stand at room temperature for 20 min. The mixed solution (200 μL) was added to the culture in each well on the plates; the cell culture plates were shaken gently before and after to mix the mixed solution and the culture medium in the culture plate. The cells were then cultured in an incubator at 37°C and 5% CO_2_. After 6 h, the mixed solution is replaced with normal medium. The culture was continued for 24 h, and then the transfection efficiency was detected using real-time fluorescence quantitative PCR (qRT-PCR) and western blot.

**TABLE 1:** Primer sequences.

Name	Primer	Sequence
NLRP1	Forward	5′- GCCCTGGAGACAAAGAATCC-3′
	Reverse	5′- AGTGGGCATCGTCAT GTGT-3′
NLRP3	Forward	5′- GATCTTCGCTGCGATCAACA-3′
	Reverse	5′- GGGATTCGAAACACGTGCATTA-3′
Caspase-1	Forward	5′- ATGCCGTGGAGAGAAACAAG-3′
	Reverse	5′- CCAGGACACATTATCTGGTG-3′
IL-1β	Forward	5′- CCAGGGACAGGATATGGAGCA-3′
	Reverse	5′- TTCAACACGCAGGACAGGTACAG-3′
GPX4	Forward	5′- GCAACCAGTTTGGGAGGCAGGAG-3′
	Reverse	5′-CCTCCATGGGACCATAGCGCTTC-3′
TFR1	Forward	5′-CCCAGGCTTCCCTTCGT-3′
	Reverse	5′-GGGCTCCAATCACAACATAC-3′
ACSL4	Forward	5′- GCTTCCTATCTGATTACCAGTGTTGA-3′
	Reverse	5′- GTCCACATAAATGATATGTTTAACACAACT-3′
GAPDH	Forward	5′-GATTTGGCCGTATCGGAC-3
	Reverse	5′-GAAGACGCCAGTAGACTC-3′
siRNA-NC	Forward	5′-UUCUCCGAACGUGUCACGUTT-3′
	Reverse	5′-ACGUGACACGUUCGGAGAATT-3′
siRNA- NLRP1	Forward	5′-CCAAAUGGCCCACUUUAAATT-3′
	Reverse	5′-UUUAAAGUGGGCCAUUUGGTT-3′

#### Overexpression of NLRP1

The NLRP1 activator muramyl dipeptide (MDP) (Selleck, United States) was added to the oxidative stress model at a concentration of 100 μmol for 24 h, in accordance with a preliminary experiment and published studies (Hsu et al., 2008; Hedl and Abraham, 2013; Bryant et al., 2017; Yi et al., 2019).

#### Western Blot

Cultured cells were harvested with a rubber scraper and washed twice with cold phosphate-buffered saline (PBS). Cell pellets were lysed and kept on ice for at least 20 min in RIPA lysis buffer (Millipore, United States) containing phenylmethylsulphonyl fluoride and a protease inhibitor cocktail (Thermo Fisher Scientific, United States). The lysates were then cleared by centrifugation and the supernatants were collected. The BCA assay was used to determine protein concentrations and 5 × loading buffer was added to each protein sample, followed by incubation for 5 min at 95°C. Then, proteins were loaded on an SDS-PAGE polyacrylamide gel, transferred to an Immobilon-P PVDF membrane (Millipore), probed with a 1:3000 dilution of all primary antibodies, and detected by chemiluminescence (ECL, Thermo Fisher Scientific). Images were then acquired by Image-Lab software (Bio-Rad, United States). Image analysis of western blots was performed with Image-Lab analyzer software.

#### Immunofluorescence

We seeded 5 × 10^5^ cells into 24-well plates on glass coverslips. Transfected cells were then grown on glass coverslips, fixed for 30 min in 4% paraformaldehyde, and then permeabilized with 0.5% Triton X-100 (Promega) in PBS buffered saline for 30 min. After washing with TBS-0.1% Triton X-100 (TBSTx), non-specific binding sites were blocked with TBSTx-5% BSA for 60 min. Then, cells were treated sequentially with an appropriate concentration of rabbit polyclonal primary antibody at 4°C overnight, and a 1:200 dilution of goat anti-rabbit IgG at room temperature for 60 min in the dark. For a negative control, PBS was added instead of the primary antibody. Immunofluorescence was subsequently visualized under a laser-scanning confocal microscope (400x) (Leica, China, SP5). The embedded uterine tissue wax pieces were sectioned with a Leica pathological slicer, and then section dewaxing, and antigen repair were performed, and serum was sealed. The following steps are the same as for cellular immunofluorescence. Immunofluorescence was subsequently visualized under a laser-scanning confocal microscope (200x) (Leica, China, SP5). Image J software was used to conduct fluorescence intensity statistics for all results.

#### qRT-PCR

Total RNA was extracted according to the manufacturer’s instructions for Trizol (Ambion, United States). The following two-step process was used to amplify the program on an ABI PRISM 7500 (ABI, United States) PCR system. First, reverse transcription of the cDNA was carried out at 25°C for 5 min, 50°C for 15 min, 85°C for 5 min, and 4°C for 10 min. Then qRT-PCR was carried out at 50°C for 2 min and 95°C for 10 min, followed by 40 cycles of 95°C for 30 s and 60°C for 30 s. The relative expression of each sample was then calculated using the 2^–ΔΔ*Ct*^ method (Li et al., 2020). PCR primers, and the primers needed for the internal control (*GAPDH*) were synthesized by TSINGKE^TM^ (China), as listed in Table 1.

#### Detection of GSH, SOD, and MDA Activities in Cell Homogenate

Culture supernatant was removed and washed twice with PBS. Then, the activities of GSH, SOD, and MDA in the cell homogenate were detected using specific kits.

### Changes in the Expression of Inflammasomes After the Overexpression and Inhibition of Ferroptosis

#### Cell Viability and the Effects of Different Concentrations of Ferroptosis Inhibitor and Activator

HTR-8/SVneo cells were seeded in 6-well culture plates (2 × 10^5^ cells/well) and cultured with different concentrations of ferrostatin-1 (a ferroptosis inhibitor; 0.1, 0.5, 1 μmol) and different concentrations of erastin (a ferroptosis activator; 1, 5, 10 μmol) for 24 h. Subsequently, we determined cell viability using an established assay. The culture supernatant was removed and washed twice with PBS, and then mixed with 110 μL of CCK-8 working solution (prepared in advance at a 1:10 volume ratio of CCK-8 solution to culture medium). This mixture was incubated at 37°C for 2 h, and then the absorbance (expressed as the optical density [OD]) value of each well at 450 nm was detected using an automatic enzyme scale. The cell survival rate was then calculated using the following formula: survival rate (%) = (experiment-group OD – blank-pore OD)/(control-group OD – blank pore OD) × 100%. An inverted phase contrast microscope was also used to observe morphology of cells in each group.

#### Expression of Inflammasomes With Changing Levels of Ferroptosis

The expression levels of NLRP1, NLRP3, caspase-1, and IL-1β were detected by western blot, qRT-PCR, and immunofluorescence, as described in section “Establishment of an Oxidative Stress Model in HTR-8/SVneo Cells.” All RNA sequences are shown in Table 1.

### Detection of IL-1β and Caspase-1 Using ELISA

The culture supernatant was extracted and coated on a porous enzyme plate with a single antibody against human IL-1β and caspase-1. The IL-1β and caspase-1 in the samples and standards were combined with the single antibody, and a biotinized anti-human antibody was added. IL-1β and caspase-1 formed an immune complex that adhered to the plate. Streptavidin (labeled by horseradish peroxidase) was then combined with biotin, and the substrate working solution turned blue. Finally, sulfuric acid was added to the termination solution, and the OD value was measured at 450 nm. The concentrations of IL-1β and caspase-1 in the same sample were directly proportional to the OD value, and could therefore be calculated by drawing a standard curve.

### Reagents and Antibodies

GPX4 (ab125066), NLRP1 (ab36852), IL-1β (ab226918), goat anti-rabbit IgG (ab150077) antibodies, and caspase-1 (ab219633) kits were obtained from Abcam (Cambridge, United Kingdom). GAPDH (10494-1-AP), NLRP3 (19771-1-AP) TFR1 (10084-2-AP) ACSL4 (22401-1-AP), and Caspase-1 (22915-1-AP) antibodies were obtained from Proteintech (Chicago, United States). GSH (S0052), SOD (S0103), MDA (S0131S), and IL-1β (PI305) kits were obtained from Beyotime (Shanghai, China). Ferrostatin-1 (HY-100579) and erastin (HY-15763) were obtained from MedChemExpress (Shanghai, China).

### Statistical Analyses

All data are expressed as the mean ± standard deviation (SD) of three independent experiments and analyzed by GraphPad Prism 9.0 (GraphPad Software, CA, United States). Statistically significant differences (^∗^*P* < 0.05, ^∗∗^*P* < 0.01, ^∗∗∗^*P* < 0.001, ^****^P < 0.0001) were identified using the Student’s *t*-test and one-way analysis of variance (ANOVA). A Bonferroni adjusted *P* < 0.05 was considered statistically significant.

## Results

### Activation of Inflammasome and Ferroptosis Were Observed in the Aborted Rats

**FIGURE 1:**
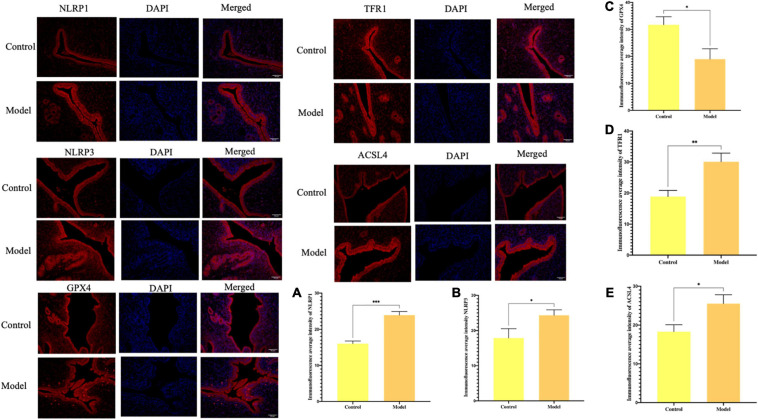
Immunofluorescence for the protein expression and % area for the immunohistochemical staining intensity in uterus tissue in rats. **(A)** The expression of NLRP1. **(B)** The expression of NLRP3. **(C)** The expression of GPX4. **(D)** The expression of TFR1. **(E)** The expression of ACSL4 (*P < 0.05, **P < 0.01, and ***P < 0.001).

According to the previous *in vitro* experiment results, we aimed to explore if ferroptosis and excessive activation of inflammasome occurs simultaneously in the uterus of aborted rats. By investigating rat abortion model, the results showed NLRP1 and NLRP3 expression in abortion rat uterus increased (^∗^*P* < 0.05, ^∗∗^*P* < 0.01), TFR1 and ACSL4 expression increased and GPX4 expression was decreased (^∗^*P* < 0.05, ^∗∗^*P* < 0.01), that compared with the control group. Indicating ferroptosis and overexpression of inflammasome did occur in the aborted rats (Figures 1A–E).

### Ferroptosis in an Oxidative Stress Model in HTR-8/SVneo Cells

**FIGURE 2:**
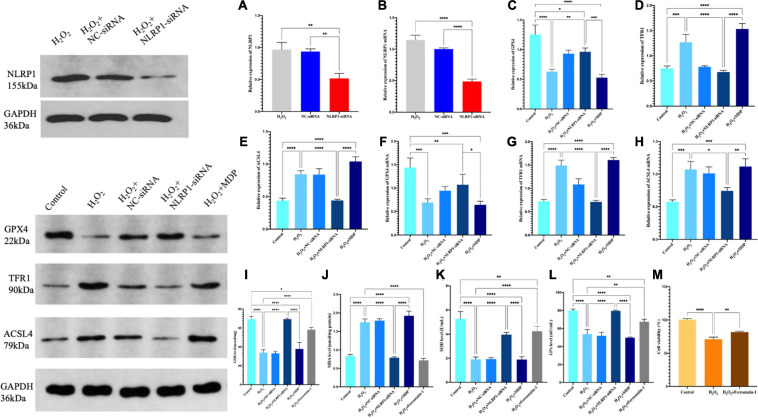
The expression levels of the NLRP1 in HTR-8/SVneo cells transfected with siRNA-NLRP1 were detected by western blot **(A)** and qRT-PCR **(B)**. The expression levels of NLRP1 at the mRNA levels were significantly down-regulated after siRNA-NLRP1 (**P < 0.01), as compared with the H_2_O_2_ and siRNA-NC groups. **(C–E)** Ferroptosis-related protein expression in HTR-8/SVneo cells were detected by western blot. **(C)** GPX4, **(D)** TFR1, and **(E)** ACSL4 (**P < 0.01). **(F–H)** mRNA expression in HTR-8/SVneo cells was detected by qRT-PCR. **(F)** GPX4, **(G)** TFR1, and **(H)** ACSL4 (*P < 0.05 and **P < 0.01). **(I–K)** Ferroptosis levels in the HTR-8/SVneo cell model of oxidative stress l. **(I)** GSH, **(J)** MDA, **(K)** SOD, and **(L)** GPx. **(M)** CCK-8 assay was used to detect cell activity (*P < 0.05, **P < 0.01, ***P < 0.001, and ****P < 0.0001). Values represent means ± SD, *n* = 3 per group.

**FIGURE 3:**
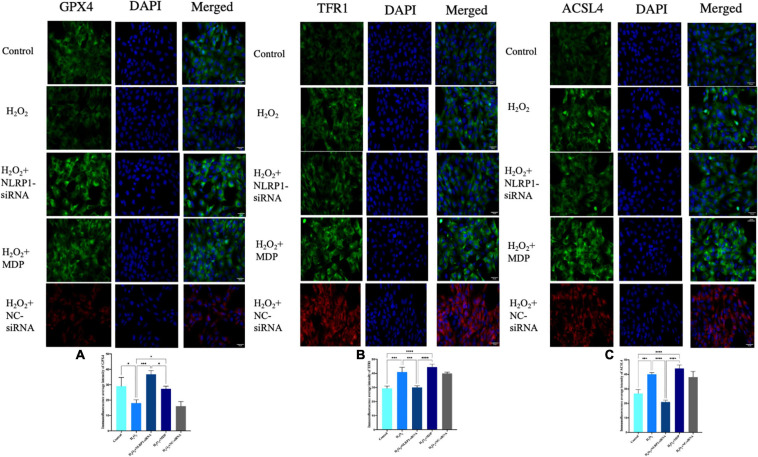
Ferroptosis-related protein expression in HTR-8/SVneo cells, as determined by immunofluorescence. Rabbit anti-GPX4 monoclonal antibody (green) and DAPI staining were used to visualize nuclear (blue) immunolabeling. Scale: 50 μm. Nuclear DAPI staining (blue) with rabbit anti-TFR1 monoclonal antibody (green) immunostaining, as shown by fluorescence microscopy. Scale: 50 μm. Nuclear DAPI staining (blue) with rabbit anti-ACSL4 monoclonal antibody (green) immunostaining, as shown by fluorescence microscopy. Scale: 50 μm. siRNA-NC (red) and DAPI staining were used to visualize nuclear (blue) immunolabeling. Scale: 50 μm. Mean immunofluorescence intensity of ferroptosis-associated proteins: **(A)** GPX4, **(B)** TFR1, and **(C)** ACSL4 (*P < 0.05, **P < 0.01, ***P < 0.001, and ****P < 0.0001). Values represent means ± SD, *n* = 3 per group.

In our previous work, we found that the NLRP1 inflammasome was overexpressed in an oxidative stress model in placental trophoblast cells (Li et al., 2020) and in the rat model of abortion that these could be used to investigate the presence of ferroptosis in an oxidative stress model. First, we used H_2_O_2_ to establish a cell model of oxidative stress and then detected the expression levels of GPX4, a key protein involved in ferroptosis, by western blot. We found that the levels of GPX4 in the model were significantly lower than that in the control group (*P* < 0.01). In contrast, the expression levels of two other key proteins (TFR1 and ACSL4) were significantly increased (*P* < 0.01) (Figures 2C–E). Ferroptosis is characterized by excessive lipid peroxidation mediated by Fe (II). To confirm that this was the case in our model, we also detected the expression levels of GSH, SOD, and MDA in cells. Compared with the control group, the expression levels of MDA in the model group after H_2_O_2_ treatment were significantly higher (*P* < 0.01), while the levels of SOD and GSH were significantly lower (*P* < 0.01) (Figures 2I–K). This difference in expression was not only detected at the protein level by western blot; the same results were evident in our analysis of cellular immunofluorescence (Figure 3) and RNA levels (Figures 2F–H) (*P* < 0.01). Therefore, both NLRP1 inflammasome and ferroptosis phenomena exist in the cellular model of oxidative stress.

### The NLRP1 Inflammasome Affects the Occurrence and Development of Ferroptosis in a Model of Oxidative Stress in HTR-8/SVneo Cells

We found that the model of oxidative stress exhibited significant activity related to the NLRP1 inflammatory corpuscle and excessive ferroptosis; however, the precise relationship between these processes remains unknown. To investigate whether the oxidative stress/ferroptosis was caused by the NLRP1inflammatory corpuscle, we first used RNAi technology to silence the expression of NLRP1 in cells; the efficiency of silencing exceeded 70% (*P* < 0.01) (Figures 2A,B).

After successfully silencing the expression of NLRP1, western blot results showed that the expression of ferroptosis had not changed. Compared with the H_2_O_2_ model group, the expression levels of GPX4 were significantly increased in the silencing group (*P* < 0.01); the expression levels of TFR1 and ASCL4 were significantly decreased (*P* < 0.01) (Figures 2C–E). The expression of ferroptosis was significantly reduced in the silencing group when compared with the H_2_O_2_ group (*P* < 0.01). The same changes were also evident at the mRNA level (Figures 2F–H). qRT-PCR results showed that levels of GPX4 mRNA were significantly increased after NLRP1 silencing when compared with the model group (*P* < 0.01), while those of TFR1 and ACSL4 were significantly decreased (*P* < 0.01). We also observed the same phenomenon in our cellular immunofluorescence analysis (*P* < 0.01) (Figure 3). In addition, we also detected the expression levels of GSH, SOD, and MDA in cells (Figures 2I–K); results showed that the levels of these three markers of ferroptosis changed accordingly after the NLRP1 inflammasome had been silenced (*P* < 0.01) and the phenomenon of ferroptosis had been alleviated. This proved that the remission of ferroptosis was changed with the NLRP1 inflammasome.

On the other hand, as shown in Figures 2, 3, to confirm the validity of this view, we added MDP, a specific activator of the NLRP1 inflammasome, into the cell model of oxidative stress to overexpress NLRP1. Western blot showed that the expression levels of GPX4 were significantly decreased compared with the control group and the silenced group (*P* < 0.01); in contrast, the expression levels of TFR1 and ACSL4 were significantly increased (*P* < 0.01), thus indicating that the phenomenon of ferroptosis had returned. Results arising from cellular immunofluorescence and qRT-PCR were consistent with the western blot results (*P* < 0.01). We also detected the expression levels of GSH, SOD, and MDA in the cells. As expected, these results also changed, indicating that ferroptosis increased with NLRP1 activation (*P* < 0.01).

### Ferroptosis Affected the Expression of the NLRP1 Inflammasome

**FIGURE 4:**
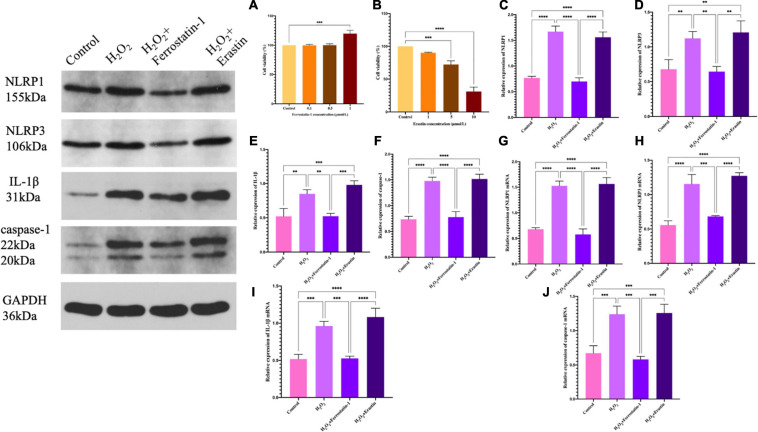
**(A)** The effect of ferrostatin-1 concentration on HTR-8/SVneo cell activity, as determined by the CCK-8 assay. **(B)** The effect of erastin concentration on HTR-8/SVneo cell activity, as determined by the CCK-8 assay. **(C–F)** Inflammasome-related protein expression in HTR-8/SVneo cells was detected by western blot. **(C)** NLRP1, **(D)** NLRP3, **(E)** IL-1β, and **(F)** caspase-1. **(G–J)** mRNA expression in HTR-8/SVneo cells was detected by qRT-PCR. **(G)** NLRP1, **(H)** NLRP3, **(I)** IL-1β, and **(J)** caspase-1 (*P < 0.05, **P < 0.01, ***P < 0.001, and ****P < 0.0001). Values represent means ± SD, *n* = 3 per group.

We found that the NLRP1 inflammasome influenced the development of ferroptosis, but this only represented a unilateral positive relationship. In order to perform a reverse test as to whether ferroptosis could also influence the NLRP1 inflammasome corpuscle, we added a ferroptosis inhibitor (ferrostatin-1) or a ferroptosis activator (erastin) to our placenta model of oxidative stress. This would provide data from two opposing angles and verify the true relationship between ferroptosis and the NLRP1 inflammasome corpuscle. Ferrostatin-1 is a lipophelic antioxidant that is effective in preventing ferroptosis, a significant non-apoptotic form of cell death caused by lipid peroxidation. Erastin, an oncogenic RAS-selective and lethal small molecule that can trigger a unique iron-dependent form of non-apoptotic cell death, which is referred to as ferroptosis. In fact, erastin, like glutamate, suppresses the uptake of the cystine/glutamate antiporter, which creates a gap in the cell’s antioxidant defense and ultimately leads to iron-dependent oxidative death. Therefore, we detected the cell viability by CCK-8. The results showed that 1 μmol of ferrostatin-1 and 1 μmol of erastin did not cause cell damage when treating HTR-8/SVneo cells (*P* < 0.01), but also achieved the purpose of inhibition or activation meaning that experiments could go-ahead efficiently (Figures 4A,B).

**FIGURE 5:**
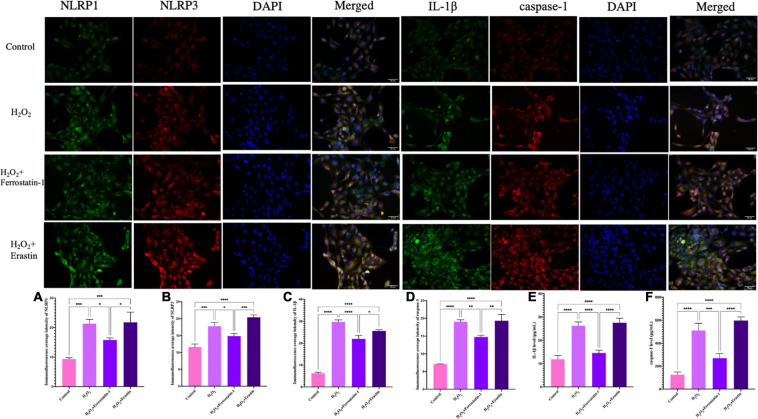
Inflammasome-related protein expression in HTR-8/SVneo cells, as determined by immunofluorescence. Rabbit anti-NLRP1 monoclonal antibody (green) and DAPI staining were used to visualize nuclear (blue) immunolabeling. Scale: 50 μm. Nuclear DAPI staining (blue) with rabbit anti-NLRP3 monoclonal antibody (red) immunostaining as shown by fluorescence microscopy. Scale: 50 μm. Inflammasome-related protein expression in HTR-8/SVneo cells, as determined by immunofluorescence. Rabbit anti-IL-1β monoclonal antibody (green) and DAPI staining were used to visualize nuclear (blue) immunolabeling. Scale: 50 μm. Nuclear DAPI staining (blue) with rabbit anti-caspase-1 monoclonal antibody (red) immunostaining, as determined by fluorescence microscopy. Scale: 50 μm. Mean immunofluorescence intensity of inflammasome-associated proteins: **(A)** NLRP1, **(B)** NLRP3, **(C)** IL-1β, and **(D)** caspase-1. **(E)** The levels of IL-1β in HTR-8/SVneo cells, as detected by ELISA. **(F)** The levels of caspase-1 level in HTR-8/SVneo cells, as detected by ELISA (*P < 0.05, **P < 0.01, ***P < 0.001, and ****P < 0.0001). Values represent means ± SD, *n* = 3 per group.

After the addition of inhibitors, we detected the expression of the NLRP1 inflammasome, NLRP3, the star protein of inflammasome family, and the expression levels of IL-1β and caspase-1, the activation products of inflammasome. Western blot showed that the expression levels of the inflammasome in the inhibitor group was significantly lower than that in the model of oxidative stress (*P* < 0.01) (Figures 4C–F); the quantification of mRNA levels also showed the same result (*P* < 0.01) (Figures 4G–J). The fluorescence intensity of the inhibitor group was significantly lower than that of the model group (*P* < 0.01) (Figure 5). Similarly, we used ELISA to detect the expression levels of inflammasome activation products in the supernatant of cell culture and found that the expression levels of IL-1β and caspase-1 in the inhibitor group were significantly lower than those in the model group (*P* < 0.01) (Figures 5E,F). On the other hand (Figures 4, 5), we also observed the effect of a specific ferroptosis activator on the inflammasome. Following the addition of the activator, western blot, qRT-PCR, and immunofluorescence results showed that the expression levels of NLRP1, NLRP3, IL-1β, and caspase-1 were significantly increased compared with the control group (*P* < 0.01) and the inhibitor group (*P* < 0.01). In addition, our ELISA results also showed the same changes (*P* < 0.01). Compared with the control group and the inhibitor group, the levels of IL-1β and caspase-1 in the activator group also showed a significant upwards trend, which was positive and intuitive (*P* < 0.01). These results may suggest that changes in the NLRP1 inflammasome also changed with alterations in ferroptosis.

## Discussion

Embryo formation is a complex process of synergistic action between the embryo and the mother. The successful implantation of the human embryo, placental formation, embryo growth and development are closely related to the proliferation, differentiation, and invasion function of the trophoblast. Excess oxidative stress is often associated with the pathology of many pregnancy-related diseases. The benefits of good ROS control in a successful pregnancy are gradually being recognized (Wang et al., 2017). Our previous studies have found that H_2_O_2_ can be used to establish an *in vitro* placental model of oxidative stress in trophocytes (Li et al., 2020). In this model, the presence of large amounts of ROS caused apoptosis and a state of oxidative stress. During this state, we also observed large amounts of the NLRP1 inflammasome; these levels increased as the state of oxidative stress increased. At the same time, we also established the oxidative stress of the abortion rat model, the results are also consistent with the *in vitro* experiment.

Ferroptosis is a newly recognized mechanism of programmed cell death that is characterized by the iron-dependent accumulation of lipid peroxides and ROS aggregation (Stockwell et al., 2017). Numerous studies have confirmed the adverse effects of ferroptosis on health and disease (Dixon et al., 2012; Hirschhorn and Stockwell, 2019). However, although oxidative stress is associated with the pathogenesis of diseases in pregnancy, there is insufficient evidence to suggest that its occurrence is associated with ferroptosis. Ferroptosis is closely related to inflammatory reactions, although previous research has mainly concentrated on tumors, cardiovascular disease, neurological diseases, research targeting pathological pregnancy and inflammatory disease in the female genitals, has been scare thus far. Furthermore, interaction between the NLRP1 inflammasome and ferroptosis in the pathogenesis of pathological pregnancy has yet to be investigated. Therefore, we aimed to explore whether ferroptosis is associated with oxidative stress in placental trophoblast cells or abortion rat model and whether ferroptosis is related to the NLRP1 inflammasome. Our experimental results showed that excessive ferroptosis occurred in trophoblast cells or abortion rat model under oxidative stress; this was manifested by low levels of SOD and GSH and high levels of MDA; as well as reduced levels of GPX4 and increased levels of TFR1 and ACSL4; in trophoblast cells.

We also aimed to identify the relationship between ferroptosis and the NLRP1 inflammasome in a model of oxidative stress in placental trophoblast cells while considering three mechanisms of ferroptosis. First, acyl-CoA synthetase long-chain family member 4 (ACSL4), a member of the long-chain acyl-CoA synthetase (ACSL) family, is an important gene in the lipid metabolism pathway. By knocking out the ACSL4 gene in mouse and human cells, the ability of erastin to induce cell mortality can be reduced effectively. Compared with ferroptosis-sensitive cells, such as HepG2 and HL60, ACSL4 expression has been shown to be significantly down-regulated in iron-death-resistant cells. CRISPR/Cas9 has also been used to perform genome-wide screening and microarray analysis; results showed that ACSL4 plays a key role in the regulation of ferroptosis *via* the lipid metabolism pathway (Doll et al., 2017). In the present study, we found that the expression of ACSL4 was increased in oxidative stress but reduced after the silencing of NLRP1. However, this change in expression was subsequently increased after the application of MDP, a specific activator of NLRP1. Secondly, amino acid metabolism is also known to be involved in the process of ferroptosis. GSH is an important antioxidant in cells and plays an important role in scavenging free radicals and maintaining redox balance, both inside and outside of cells (Wu et al., 2019). Glutathione peroxidase 4 (GPX4) is a dependent GSH enzyme that can reduce glutathione into oxidized glutathione (GSSG), the lipid hydroperoxide (L-OOH) reduction of fatty alcohols (L-OH), or convert free H_2_O_2_ into water, against relying on the lipid peroxidation of iron and O_2_, protecting cell membrane structure and the function is not an oxide interference and damage, and the lack of GSH will cause GPX4 function is impaired, promoting ferroptosis (Sun et al., 2018). Erastin inhibits GPX4 activity by reducing GSH synthesis (Liang et al., 2019), thereby promoting the accumulation of ROS (Yang et al., 2014). GPX4 inhibits ferroptosis by scavenging lipid peroxides in cells, inhibits the expression or activity of GPX, and promotes the occurrence of ferroptosis in cells (Yang et al., 2014; An et al., 2019). Studies have shown that the overexpression of GPX4 inhibits the accumulation of intracellular ROS (Shen et al., 2021), and that lipid peroxidation and intracellular ROS accumulation can be promoted by the knockout of membrane lipase GPX4 or the direct use of a GPX4 inhibitor (Yang et al., 2014). Animal experiments have also shown that GPX4 knockout mice exhibit ferroptosis in the renal tubule cells (Kagan et al., 2020). Therefore, GPX4 may be a key regulator of ferroptosis. In order to prove this point, we detected the expression of GSH and GPX4 after the silencing of NLRP1 and found that the expression of GSH and GPX4 were increased. Then we activated NLRP1 and found that the expression levels of both GSH and GPX4 were decreased. Inflammation is a basic pathological process in which the body is acts defensively to the stimulation of various injury factors. Ferroptosis is closely related to the inflammatory response, during which a variety of inflammatory factors are produced that are related to peroxide metabolism. GPX4 reduces ROS levels and alleviates inflammatory responses (Tsurusaki et al., 2019). Once GPX4 is deficient, an appropriate inflammatory response cannot be induced (Wang et al., 2020). Our results are consistent with previous studies (Vaka et al., 2018). The circulating iron content mainly consists of ferric ion (Fe^3+^) which exists in a form that is bound with transferrin and enters cells *via* the membrane transferrin receptor 1 (TFR1) and endocytosis, thus forming endosomes. Previous research showed that ferroptosis-sensitive cells featuring a Ras mutation exhibited high levels of TFR1 expression and reduced expression levels of iron-storage ferritin (Yang and Stockwell, 2008), suggesting that an overload of iron, caused by increased iron uptake and reduced iron storage, contributes to the process of ferroptosis (Dixon et al., 2012). Therefore, the regulation of cellular iron uptake, storage, and utilization is an important aspect in the regulation of ferroptosis. We also examined the expression of TFR1 within this pathway and found that the silencing of NLRP1 reduced the expression levels of TFR1; in contrast, the activation of NLRP1 increased the expression levels of TFR1. These results suggest that iron-dependent lipid peroxides accumulate in the placenta under conditions of oxidative stress. Lipid peroxidation plays a key role in the dysfunction and death of trophoblast cells; this key role changes with changes that affect the NLRP1 inflammasome.

Malondialdehyde (MDA) is the oxidative end-product of intracellular lipid peroxidation and represents a marker of the oxidative state of cells. Some studies have shown that the level of serum MDA is significantly increased in patients with threatened abortion and preeclampsia (Patil et al., 2009; Ahmadi et al., 2012). Superoxide dismutase (SOD) is an important indicator that reflects the antioxidant capacity of cells. We measured the levels of MDA and SOD in cells and found a reduction in ferroptosis levels after NLRP1 silencing that was alleviated after the administration of an NLRP1 activator; these results are similar to those published previously. Therefore, SOD activity and MAD levels can indirectly reflect the level of cellular oxidative stress and the intensity of ferroptosis (Ma et al., 2016; Fujii et al., 2019).

In view these findings, it is evident that we need to explore the effect of ferroptosis on NLRP1 inflammasome. Ferrostin-1 is a first-generation inhibitor of ferroptosis that can effectively inhibit the process of ferroptosis by inhibiting lipid peroxidation *in vitro* (Jiang et al., 2015). The small molecule erastin has been shown to increase the consumption of GSH in cells and deactivate GPX4, causing the accumulation of lipid peroxidation and ferroptosis in cells (Yang et al., 2014) and representing a classical activator of ferroptosis (Dixon et al., 2012). We applied an inhibitor (ferrostin-1) and activator (erastin) of ferroptosis, after having determined the optimal concentrations. We then examined how the NLRP1 inflammasome changed in response to these agents. We found that the presence of ferrostin-1 led to a reduction in the expression of NLRP1, NLRP3, IL-1β, and caspase-1 expression in placental trophoblast cells, indicating that the inflammatory state was suppressed. During a state of chronic inflammation, the NLRP1 and NLRP3 inflammasomes are overactivated over a long period of time, constantly transforming into active caspase-1 and promoting the release of IL-1β; the release of IL-1β can lead to the occurrence and maintenance of inflammation (Miao et al., 2010). Caspase-1, as a factor associated with the inflammasome, can promote the production of apoptotic proteins and apoptotic factors and can also increase injury as a result of oxidative stress. In addition, studies have shown that the activation of the NLRP3 inflammasome can rapidly produce IL-1β (Swaroop et al., 2018). Therefore, the NLRP1 and NLRP3 inflammasome signaling pathways may be involved in the regulation of mechanisms associated with pathological pregnancy by up-regulating the expression levels of factors related to caspase-1 and IL-1β, thus influencing the progression of disease. However, in a state of ferroptosis overactivation, these inflammasomes are also overexpressed, thus exacerbating the inflammatory state. These results all suggest that the NLRP1 inflammasome also changes with the changing levels of ferroptosis.

There are still some limitations in this study, as further *in vivo* and pre-clinical investigations is warranted in the future. In-depth studies relating to the regulation of the NLRP1 inflammasome and ferroptosis in trophoblasts will help us to elucidate the pathogenesis of a class of primary trophoblastic diseases such as abortion, preeclampsia, and fetal growth restriction, and provide new therapeutic targets for the treatment of these diseases.

## Conclusion

**FIGURE 6:**
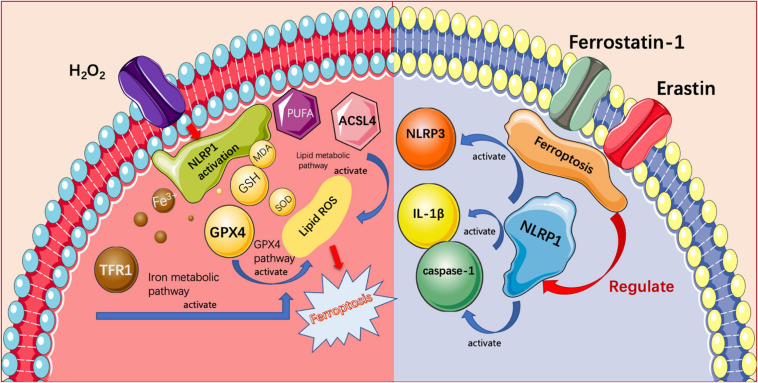
NLRP1 causes ferroptosis in trophoblast cells through three pathways and ferrostatin-1 and erastin regulate the expression of NLRP1 inflammasome in trophoblast cells by affecting ferroptosis.

In summary, as shown in Figure 6, the cascading relationship between ferroptosis and the NLRP1 inflammasome was investigated, for the first time, by using a model of oxidative stress in placental trophoblasts. We demonstrated that ferroptosis and inflammasome activation were observed in the oxidative stress induced abortion rat model. To verify the relationship between these, we conducted cytological experiments. The ferroptosis was evident in trophoblast cells under oxidative stress, and that the extent of ferroptosis was reduced after silencing the NLRP1 inflammasome by siRNA-NLRP1. The application of the NLRP1 activator led to an increase in ferroptosis. Furthermore, the application of an inhibitor and activator of ferroptosis demonstrated that changes in the NLRP1 inflammasome are associated with changes in ferroptosis, and that there was a mutually restrictive and interactive relationship between these two processes. This is the first study to demonstrate the association between ferroptosis and NLRP1 inflammasomes in the pathogenesis of pathological pregnancy. Our findings may provide a useful therapeutic target for the pathogenesis of pregnancy-related diseases.

## Data Availability Statement

The raw data supporting the conclusions of this article will be made available by the authors, without undue reservation.

## Ethics Statement

The animal study was reviewed and approved by the Ethics Committee of Xi’an Jiaotong University (Approval Document No. 2019-1232).

## Author Contributions

LM and GS conceived and conceptualized the project, acquired data, and wrote the draft manuscript. KM and AP were responsible for methodology and software. DH was responsible for visualization, investigation, and supervision. ZJ was responsible for writing, reviewing and editing. All authors read, edited several draft versions, and approved the final manuscript.

## Conflict of Interest

The authors declare that the research was conducted in the absence of any commercial or financial relationships that could be construed as a potential conflict of interest.

## Publisher’s Note

All claims expressed in this article are solely those of the authors and do not necessarily represent those of their affiliated organizations, or those of the publisher, the editors and the reviewers. Any product that may be evaluated in this article, or claim that may be made by its manufacturer, is not guaranteed or endorsed by the publisher.

## Abbreviations

ACSL4: Acyl-CoA synthetase long-chain family member 4
ACSL: long-chain acyl-CoA synthetase
FBS: Fetal bovine serum
GSH: glutathione peroxidase
GPX4: Glutathione peroxidase 4
GSSG: oxidized glutathione
MDA: Malondialdehyde
MDP: muramyl dipeptide
NLRP1: Nod-like receptor family protein 1
qRT-PCR: real-time fluorescence quantitative PCR
PBS: Phosphate buffer saline
ROS: Reactive oxygen species
SOD: Superoxide dismutase
TFR1: transferrin receptor 1.
